# Prediction of Cacao (*Theobroma cacao*) Resistance to *Moniliophthora* spp. Diseases via Genome-Wide Association Analysis and Genomic Selection

**DOI:** 10.3389/fpls.2018.00343

**Published:** 2018-03-20

**Authors:** Michel S. McElroy, Alberto J. R. Navarro, Guiliana Mustiga, Conrad Stack, Salvador Gezan, Geover Peña, Widem Sarabia, Diego Saquicela, Ignacio Sotomayor, Gavin M. Douglas, Zoë Migicovsky, Freddy Amores, Omar Tarqui, Sean Myles, Juan C. Motamayor

**Affiliations:** ^1^Department of Plant, Food and Environmental Sciences, Faculty of Agriculture, Dalhousie University, Truro, NS, Canada; ^2^MARS, Incorporated c/o United States Department of Agriculture – Agricultural Research Service, Miami, FL, United States; ^3^School of Forest Resources and Conservation, College of Agricultural and Life Sciences, University of Florida, Gainesville, FL, United States; ^4^Instituto Nacional de Investigaciones Agropecuarias, Quito, Ecuador; ^5^Department of Microbiology and Immunology, Faculty of Medicine, Dalhousie University, Halifax, NS, Canada; ^6^Facultad de Ciencias Agrarias, Universidad Técnica Estatal de Quevedo, Quevedo, Ecuador

**Keywords:** *Theobroma cacao*, witches’ broom disease, frosty pod rot, SNPs, GWAS, genomic selection

## Abstract

Cacao (*Theobroma cacao*) is a globally important crop, and its yield is severely restricted by disease. Two of the most damaging diseases, witches’ broom disease (WBD) and frosty pod rot disease (FPRD), are caused by a pair of related fungi: *Moniliophthora perniciosa* and *Moniliophthora roreri*, respectively. Resistant cultivars are the most effective long-term strategy to address *Moniliophthora* diseases, but efficiently generating resistant and productive new cultivars will require robust methods for screening germplasm before field testing. Marker-assisted selection (MAS) and genomic selection (GS) provide two potential avenues for predicting the performance of new genotypes, potentially increasing the selection gain per unit time. To test the effectiveness of these two approaches, we performed a genome-wide association study (GWAS) and GS on three related populations of cacao in Ecuador genotyped with a 15K single nucleotide polymorphism (SNP) microarray for three measures of WBD infection (vegetative broom, cushion broom, and chirimoya pod), one of FPRD (monilia pod) and two productivity traits (total fresh weight of pods and % healthy pods produced). GWAS yielded several SNPs associated with disease resistance in each population, but none were significantly correlated with the same trait in other populations. Genomic selection, using one population as a training set to estimate the phenotypes of the remaining two (composed of different families), varied among traits, from a mean prediction accuracy of 0.46 (vegetative broom) to 0.15 (monilia pod), and varied between training populations. Simulations demonstrated that selecting seedlings using GWAS markers alone generates no improvement over selecting at random, but that GS improves the selection process significantly. Our results suggest that the GWAS markers discovered here are not sufficiently predictive across diverse germplasm to be useful for MAS, but that using all markers in a GS framework holds substantial promise in accelerating disease-resistance in cacao.

## Introduction

Cacao (*Theobroma cacao*) is tropical understory tree native to the Amazon basin that produces one of the world’s most valuable agricultural commodities: cacao beans. As the primary ingredient in chocolate, cacao trees are the base of a $100 billion USD global industry (World Cocoa Foundation, 2012) and a substantial contributor to the economies of West Africa and Latin America (Franzen and Mulder, 2007). Yields can be as high as 3,000 kg ha^-1^, but pathogens severely limit production: as much as 30% of the crop is estimated to be lost annually due to disease (Bowers et al., 2001). The majority of these losses come from three fungal pathogens, dubbed the ‘cacao disease trilogy’ (Fulton, 1989; Evans, 2007): black pod rot (BPR), witches’ broom disease (WBD), and frosty pod rot disease (FPRD). Although BPR is by far the most serious pathogen in terms of annual losses, WBD and FPRD may have the potential to be even more damaging due to the fact they have not yet spread to West Africa, the largest center of cacao production (Ploetz, 2007).

Both WBD and FPRD are caused by basidiomycete fungi (*Moniliophthora perniciosa* and *M. roreri*, respectively), which are closely related (Aime and Phillips-Mora, 2005; Meinhardt et al., 2014). Both fungi have co-evolved with cacao and related species in its native range and have spread throughout the Americas (Evans, 2007; Evans et al., 2013). WBD colonizes meristematic tissue, and can infect shoots, flowers and developing fruit, sometimes resulting in the death of the entire tree (Meinhardt et al., 2008). FPRD only infects pods, but its aggressiveness and persistence has resulted in the abandonment of cacao cultivation in large areas in the Americas (Phillips-Mora and Wilkinson, 2007). Current methods for controlling these diseases center on the application of fungicides/biocontrol agents and phytosanitation practices on-site, and the restriction of movement of the pathogens to new areas that are not yet affected (Bowers et al., 2001; Ploetz, 2016). These strategies, however, are considered ‘short- to medium-term’ (Hebbar, 2007); long-term solutions will require the development of disease-resistant germplasm.

Cacao is a long-lived woody perennial with an extended juvenile phase, and thus stands to benefit more than most crops from marker-assisted breeding (MAB; McClure et al., 2014). The first step toward MAB for resistance to *Moniliophthora* is the identification of genetic markers that robustly predict resistance. Thus far, studies into the development of cacao disease markers have relied on bi-parental linkage mapping (Lanaud et al., 2009; Motilal et al., 2016; Royaert et al., 2016). Although a powerful tool, this method relies on creating segregating populations from crosses, a challenging task in slow-growing perennials. Furthermore, markers identified may not be effective outside of the mapping population.

Alternatives to traditional QTL mapping include genome-wide association studies (GWAS), and genomic selection (GS). Although these two methods generally rely on more intense genotyping of single nucleotide polymorphisms (SNPs) either through next-generation sequencing (NGS; Davey et al., 2011) or high-density SNP microarrays (Gupta et al., 2008), they are generally considered more robust. GWAS functions by testing the association between phenotypes and individual SNPs in a population, generating single markers that can be used to screen germplasm for useful traits (Korte and Farlow, 2013). Conversely, GS calculates the association between phenotypes and the entire marker set within a ‘training population’ to create a model that can then be used to predict the phenotypes of individuals in a test population (Meuwissen et al., 2001; Hayes et al., 2009). Although both methods can be effective tools in MAB, the merits of each have been debated, with some suggesting that integrating the two may hold the key to better phenotype prediction (Zhang et al., 2014; Bian and Holland, 2017).

Fungal diseases, and *Moniliophthora* species in particular, remain one of the primary constraints of cacao production in the Americas and, if they extend beyond their current range, threaten to seriously damage the chocolate industry worldwide. Genetic resistance to these diseases is therefore a top priority for *T. cacao* breeders and a central focus for genomic research on this crop. The ability to accurately screen for disease resistance genetically, without having to phenotype trees at a mature stage, could greatly increase the efficiency of cacao improvement. With a choice of methods at breeder’s disposal, it is important to evaluate the effectiveness of each approach. This study aims to gauge the effectiveness of GWAS and GS in three cacao populations, mainly including selections from 225 bi-parental crosses, in predicting resistance to FRPD and WBD, as well as productivity. In addition, we seek to determine how these techniques may be applied in disease resistance prediction between related populations.

## Materials and Methods

### Populations

Crosses were made according to three factorial mating schemes according to the genetic types of the parents (**Supplementary Table S1**), including (A) wild parents (wild accessions never tested in crosses), (B) known accessions (previously selected accessions and some previously tested as parents), and (C) accessions from the ‘Nacional’ genetic group. Approximately 100 progenies were obtained from each cross, half of which were randomly selected and planted in large bags for 1–2 years (two to three rainy seasons) under mature cocoa trees highly infected with WBD, in five randomized blocks containing 10 plants each. Of these fifty, plants were selected if (a) they showed an absence of witches’ brooms symptoms, or (b) the diameter of the broom relative to the diameter of the stem from where the broom was growing of less than 0.6, a common technique for screening plants for WBD resistance at the seeding stage (Surujdeo-Maharaj et al., 2003). Further to these selections, approximately ten percent of the plants were also chosen randomly without taking into account any WBD symptoms. Individual accessions were cloned (through grafting on IMC 67 open pollinated seedlings) and planted in three adjacent plots at a test site in Ecuador (“Estacion Tropical Experimental de Pichilingue,” Rios Province, Ecuador) starting in 2007, 2009 and 2010. Plots were planted sequentially by year. For each population, three replicates were planted in four blocks (a total of *N* = 12 replicates for each clone), with the exception of Malvinas, in which only three blocks were planted (*N* = 9). Trees that died during the trial were replanted.

We therefore examined a total of 1,345 accessions from the three plots (referred to here as populations): Las Tecas’ (*N* = 589) ‘Malvinas’ (*N* = 385) and ‘Ganaderia’ (*N* = 391) were genotyped using the 15K *Theobroma cacao* L. SNP array (Livingstone et al., 2017). Although the three populations were derived from many of the same parents, the accessions in each population were largely distinct, sharing only a couple accessions between them.

### Phenotypic Data

Phenotypic observations were taken approximately every month, and aggregated per year from the year following planting until 2013. Observations for WBD traits (vegetative brooms, flower cushion broom, and chirimoya pods) were taken once per year in July. The following observations were recorded:

• Chirimoya pods: counts of developing pods infected by WBD (*Moniliophthora perniciosa*)
• Flower cushion broom: counts of cushion flowers infected by WBD
• Vegetative brooms: counts of twigs/branches infected by WBD
• Monilia pods: counts of pods infected by FPRD (*Moniliophthora roreri*)
• Healthy pods: counts of pods not infected by any pathogen
• Total pod number: includes counts of healthy pods, pods infected with FPRD, and sick pods (pods infected with pathogens not including FPRD)
• Total fresh weight of pods (g)

All phenotypes were log-transformed, apart from monilia pods and healthy pods, which were taken as a percentage of total pods. To obtain adjusted means across replicates for each genotype, the following mixed linear model was applied:

(1) y = μ+G+A+A×N+A×G+B×A×N+R+I+ε

where *y* is the phenotypic value of the accession, *μ* is the overall mean, *G* is the fixed effect of accession identity, *A* is the random effect of tree age, *N* is random effect of the year of the observation, *B* is the random block effect, *R* is the random effect of rep, *I* is the random effect of individual tree, and 𝜀 is the residual error. The adjusted value for each trait for each accession (i.e., *μ* + *G*) was used for all downstream analyses (adjusted accession values given in **Supplementary Table S2**).

### DNA Extraction and Microarray

Leaf samples were collected from the 1465 accessions at INIAP, Ecuador. The DNA from these samples was extracted using the Zymo Research plant DNA extraction kit following the manufacturer’s protocol (Zymo) and submitted to Illumina for genotyping on the custom Infinium II BeadArray. Details of the 15K SNP array are described in Livingstone et al. (2017).

### Genotypic Data

Genetic data were filtered using PLINK v1.07 (Purcell et al., 2007). The minor allele frequency threshold was set at 5% and the missingness by individual filter at 10%. Missing genotypes were imputed using LinkImpute v 1.1.1, a *k*-nearest neighbor imputation technique (Money et al., 2015). Accuracy of the imputation was 0.966 using two nearest neighbors (*k* = 2) and 65 SNPs (*l* = 65). The final genotype set, after manual curation to remove genetically identical and likely mislabeled individuals, was 1,345 accessions (Ganaderia = 391, Malvinas = 385, Las Tecas = 589, with 17 accessions common to more than one population) with a complete set of 9,640 SNPs.

### Population Structure, Ancestry, and Linkage Disequilibrium

The proportion of membership in each of the 10 cacao ancestral genetic groups (Motamayor et al., 2008) was estimated using the software Admixture (Alexander et al., 2009). Supervised admixture analysis was performed using the individuals with >0.85 proportion ancestry from a study of 200 *T. cacao* genomes (Cornejo et al., unpublished) and individuals used to describe the ancestral types (Motamayor et al., 2008) as references. Principal component analysis (PCA) of the genotype matrix was performed in R using the ‘prcomp’ function (R Core Team, 2016). Linkage disequilibrium (LD) was calculated using PLINK v1.07 (Purcell et al., 2007).

### Genome-Wide Association Analysis

Genome-wide association analysis was performed using Tassel v5.2.35 (Bradbury et al., 2007), correcting for kinship using the internally generated an identity-by-state (IBS) *k*-matrix and genetic structure (*Q*) using the ancestry estimates generated in the admixture analysis. Each population was analyzed separately using adjusted accession means (see section Phenotypic Data) as phenotypes. The *P*-value threshold for multiple tests was set as the Bonferroni correction of the effective number of independent tests (*M*_eff_; Cheverud, 2001) based on the number of principal components required to explain 99.5% of the variation observed in the SNP data.

### Genomic Prediction

Genomic prediction of phenotypes was performed using a G-BLUP model in ASREML-R (Butler et al., 2007), a mixed model with the following form:

(2) y = Xβ+Zu+ε

where *y* is the phenotypic values, *β* is the vector of fixed effects (including the intercept and, when used, single markers identified by GWAS as being significantly associated with the trait being tested) with corresponding design matrix (*X); u* is the vector of random genotypic effects, with its corresponding design matrix (*Z*), and *u*∼MVN(0, σ^2^*_u_G*), where *G* is the *k*-matrix obtained by GenoMatrix (Nazarian and Gezan, 2016) from the IBS matrix generated by Tassel; and *𝜀* is the vector of residuals, where *𝜀*∼MVN(0, σ^2^*I*), where *I* is an identity matrix. Also, σ^2^*_u_* and σ^2^ are the variance component associated with genotypic and residual effects, respectively. The narrow-sense heritability, *h*^2^, was calculated from the estimated variance components by using the following expression: *h*^2^ = σ^2^*_u_*/(σ^2^*_u_* + σ^2^).

Each of the three populations was used as a training set to generate a predictive model using all genotype/phenotype data (i.e., without cross validation) that was applied to remaining two ‘test’ populations to generate genomic-estimated breeding values (GEBVs). The accuracy of the prediction model was calculated by determining the correlation between predicted and estimated phenotypic (see section Phenotypic Data) values per accession in the test populations. To determine the general accuracy of the model, the correlation between the predicted and observed values in the training population were also calculated and reported.

Type ‘B’ correlations (Yamada, 1962), which measure the correlations between genetic values estimated with genomic prediction models in different environments, were calculated for the three populations to evaluate differences between their respective plot areas. Note that values of Type ‘B’ correlation close to zero (or one) indicate large (or small) presence of genotype-by-environment interactions, respectively.

### Selection Simulation

For a subset of traits (vegetative broom, cushion broom, monilia pod and total fresh weight), a screening trial simulation was set up to determine the effectiveness of applying different methods of selecting the top-performing genotypes at the seedling stage. For the simulation, phenotypic and genotypic information for Las Tecas, the largest population, was used as a training population to select the top 40 performing individuals (approximately 10% selection intensity) for all three populations (Las Tecas, Ganaderia, and Malvinas). Five methods were considered:

(i) *GWAS markers*, in which individuals were ranked according to the total number of SNP markers with ‘favorable’ alleles discovered in the training population they carried (in the case of ‘ties’ exceeding the 40-individual limit, individuals of the lowest rank were selected at random until the limit was reached).
(ii) *Bi-parental QTL markers*, in which individuals were ranked according to a similar scheme as (i), but using SNP markers discovered in a related bi-parental population (Livingstone et al., 2017). As markers were only available for monilia pod resistance and pod fresh weight, only these traits were considered for this method.
(iii) *Genomic selection*, in which individuals were ranked according to the predicted phenotypic values (i.e., GEBV) from the genomic prediction models generated in the training population (Las Tecas).
(iv) *Genomic selection with GWAS markers*, in which individuals in the test populations were ranked in a similar model as (iii), while using the markers in (i) as fixed effects (Bian and Holland, 2017).
(v) *Genomic selection with bi-parental QTL markers*, in which individuals in the test populations were ranked in a similar model as (iv), but using the SNP markers from (ii) as fixed effects. As in (ii), only monilia pod and pod fresh weight were considered for this method.

In addition, the Las Tecas accessions were ranked according to the seedling broom width score (see section Phenotypic Data) to determine how the genetic methods compared to an early phenotypic screening technique.

The mean phenotypic value of the top 40 individuals selected by each method for each trait was determined and compared to a distribution of means from 10,000 sets of 40-individual groups selected at random from each population (with replacement). Those selected means that fell within the top 1% of the distribution (*P* < 0.01) were considered to be significantly more favorable than choosing at random; those that fell outside the distribution (*P* < 0.0001) were considered highly significant.

## Results

### Phenotype Associations

**FIGURE 1:**
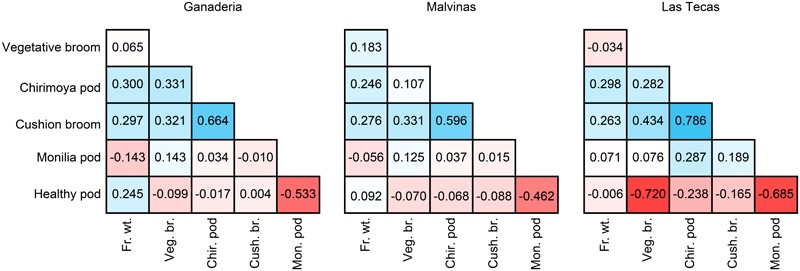
Correlation (*R*-values) between phenotypes in three cacao populations. Phenotypes were log-transformed (Veg. br., Chir. pod, Cus. br., Fr. wt.) or set as proportion of total pods (Mon. pod, Hea. pod), then adjusted using site, year, and plant age to get a mean value per genotype.

Correlations between the adjusted means of phenotypes are shown in **Figure 1**. Correlations of all phenotypes remained largely similar across all populations. All WBD phenotypic traits (vegetative broom, chirimoya pods, and cushion broom) were positively correlated, particularly the latter two. Correlations between monilia pod incidence (FPRD) and WBD disease were not as pronounced and varied across populations. Disease phenotypes did not show strong correlations with productivity measurements (total fresh weight and percent healthy pods), apart from healthy pods and monilia pods, which were negatively correlated.

### Structure and Diversity of Populations

**FIGURE 2:**
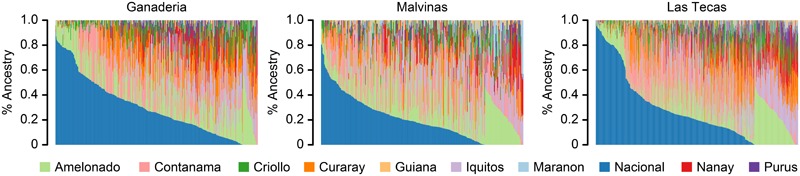
Ancestry proportions for 1,345 accessions from three cacao populations. Each accession is represented by a vertical line and derives its ancestry from up to 10 ancestral groups which are indicated by the various colors in the legend. Ancestry was estimated using supervised Admixture analysis using a genome-wide panel of 9,640 SNPs.

The percent ancestry of each of the populations is given in **Table 1** and **Figure 2**. All populations were prevailingly of ‘Nacional’ ancestry, with a mean ancestry proportion of 29%, 19%, and 26% for Ganaderia, Malvinas, and Las Tecas, respectively. This was followed by the ‘Amelonado’ (16%, 20%, and 15%) and ‘Contamana’ (14%, 17%, and 15%).

**Table 1:** Mean percent ancestries of three populations of cacao.

Ancestral Group	Ganaderia	Malvinas	Las Tecas	All
Nacional	29.7%	19.8%	26.4%	25.3%
Amelonado	15.8%	19.8%	14.6%	16.7%
Contenama	13.5%	17.0%	14.6%	15.0%
Iquitos	10.7%	11.6%	13.1%	11.8%
Curaray	12.9%	8.2%	11.0%	10.7%
Nanay	6.1%	7.2%	7.0%	6.8%
Criollo	6.3%	6.3%	6.4%	6.3%
Purus	3.2%	2.9%	3.4%	3.1%
Maranon	1.1%	5.8%	2.2%	3.0%
Guiana	0.7%	1.6%	1.3%	1.2%

**FIGURE 3:**
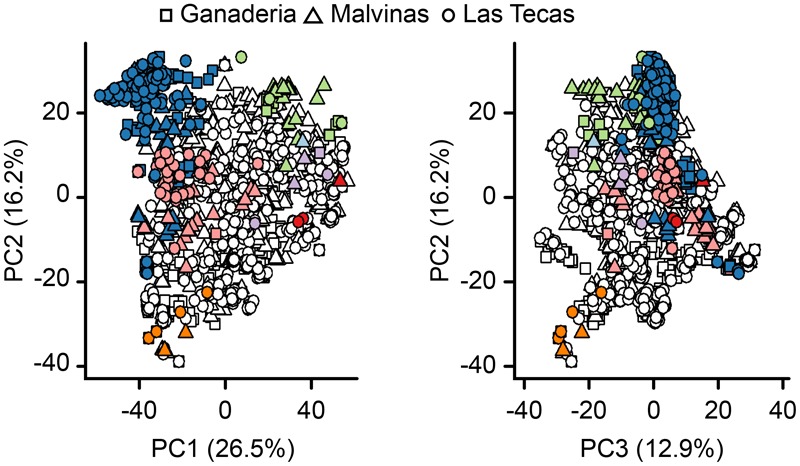
Principal component analysis (PCA) of genetic relatedness of 1,345 cacao individuals in three sites using a genome-wide panel of 9,640 SNPs. Shapes refer to the population of the individual. Colored points are individuals showing >0.5 proportion ancestry of an ancestral group (see **Figure 2** for description). Percentage of the variation captured by each component is given on the axis labels.

Principal component analysis (**Figure 3**) confirmed that the major dimensions of genetic variation were influenced by ancestral background, with ‘Nacional’/‘Contanama’-derived accessions differentiating from ‘Amelonado’/‘Iquitos’/‘Nanay’ accessions along the primary axis, and the ‘Curaray’-derived accessions separating out along the secondary axis. This analysis also suggests that the three populations, Ganaderia, Malvinas, and Las Tecas, although composed of different families are not fundamentally genetically distinct from each other, as the accessions from each population are distributed evenly among the first three PCs, which account for 55.6% of the genetic variation.

**FIGURE 4:**
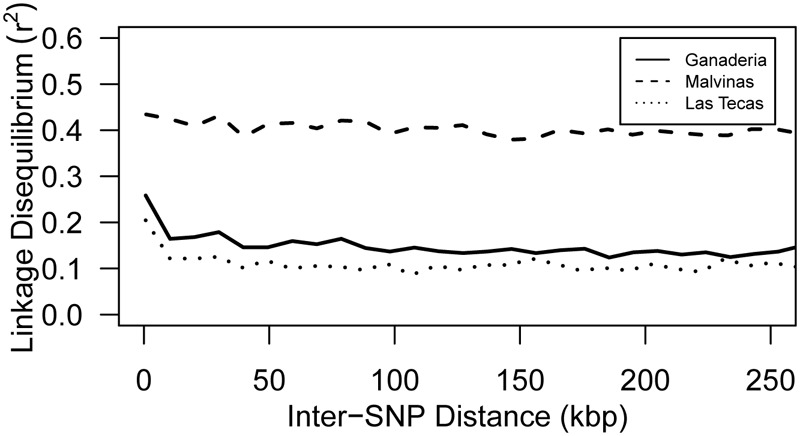
Mean pairwise SNP intra-chromosomal linkage disequilibrium (LD) by inter-SNP distance for three populations of cacao. Lines represent Loess-smoothed averages.

In contrast to ancestry and PCA, linkage disequilibrium breakdown did show some important distinctions between populations (**Figure 4**). Ganaderia and Las Tecas had an average within-chromosome LD *r*^2^ value of 0.188 and 0.147 (within a 100 kbp window), respectively, whereas Malvinas (showing a more even composition of the main genetic groups) had a mean value of 0.418, showing much less recombination.

### Genome-Wide Association Analysis

**FIGURE 5:**
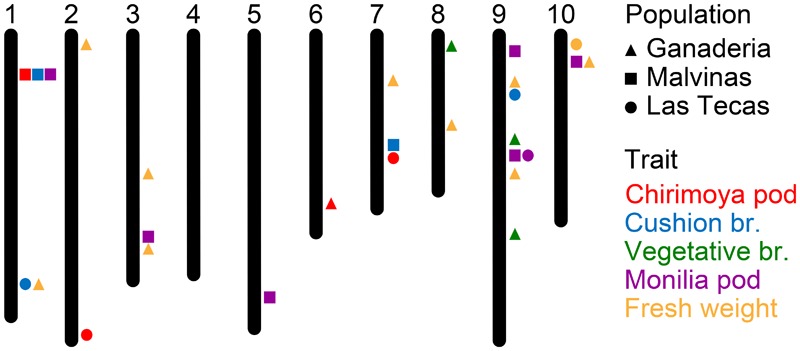
Genomic position of SNP markers significantly associated with five phenotypes among three populations of cacao (see **Supplementary Table S3** for SNP information).

Results of GWAS for the four disease traits and two productivity traits are given in **Figure 5** and **Supplementary Table S3**. Overall, only three pairs of SNPs in close proximity (>100 kbp) were shared across two populations, and only one associated with the same trait. No markers in any population were associated significantly with the percent of healthy pods.

Ganaderia had a large number of significant associations (9) associated with total fresh weight, some of which corresponded to disease markers in other populations. This population also had a significant marker for cushion broom on chromosome 6, and three hits for vegetative broom on chromosomes 8 and 9.

Malvinas had several significant associations (6) for monilia pod, spread over five chromosomes, including one on chromosome 9 that lay in relatively close proximity (∼750 kbp) to a similar marker found in Las Tecas. One ∼300 kbp region in chromosome 1 had significant associations for chirimoya pod, cushion broom and monilia pod, and another for chirimoya pod was found on chromosome 7.

Las Tecas had the fewest number of total significant associations (9) with three for chirimoya pod, two for cushion broom and one each for monilia pod and fresh weight. Most markers associated with disease phenotypes discovered in this population tended to be in regions near significant associations in the two other populations, although one marker for chirimoya pod on chromosome 2 was not found elsewhere.

### Genomic Prediction

Prediction of phenotypes via a genomic prediction model was performed using each population as a training population for the remaining two, as well as themselves (**Table 2**). Model-derived narrow-sense heritability (*h*^2^) varied greatly between traits and sites, with vegetative broom and total fresh weight showing some of the highest values, and pod diseases (chirimoya and monilia) some of the lowest. Likewise, the prediction accuracy of the models varied between traits, although they remained notably consistent between populations (the exception being healthy pods, which was predicted much less accurately using Malvinas as a test population).

**Table 2:** Accuracy of genomic selection (GS) models for six traits in three populations of cacao, using one of three populations as the training set and the remaining two as test sets.

Trait	Training population	Model *h*^2^	Test populations			Mean accuracy^∗^
			Ganaderia	Malvinas	Las Tecas	
**Vegetative broom**	Ganaderia	0.568	0.856	0.304	0.545	0.425
	Malvinas	0.193	0.450	0.611	0.505	0.478
	Las Tecas	0.675	0.578	0.376	0.889	0.477
**Chirimoya pods**	Ganaderia	0.083	0.458	0.158	0.098	0.128
	Malvinas	0.222	0.161	0.583	0.235	0.198
	Las Tecas	0.276	0.116	0.236	0.649	0.176
**Cushion broom**	Ganaderia	0.205	0.259	0.195	0.218	0.207
	Malvinas	0.219	0.171	0.643	0.202	0.198
	Las Tecas	0.238	0.159	0.298	0.655	0.229
**Monilia pods**	Ganaderia	0.219	0.620	0.070	0.245	0.158
	Malvinas	0.030	0.121	0.373	0.377	0.065
	Las Tecas	0.282	0.259	0.130	0.661	0.237
**Healthy pods**	Ganaderia	0.201	0.604	0.064	0.336	0.200
	Malvinas	0.122	0.005	0.572	0.125	0.065
	Las Tecas	0.426	0.317	0.157	0.755	0.237
**Total fresh weight**	Ganaderia	0.424	0.783	0.301	0.477	0.389
	Malvinas	0.456	0.272	0.804	0.460	0.366
	Las Tecas	0.433	0.388	0.393	0.788	0.391

*^∗^Excluding prediction of training population (i.e., diagonals). The trait narrow-sense heritability (*h*^*2*^) is given for each model. Model accuracy is defined as the correlation between predicted and observed values. Mean accuracy is the average accuracy across test populations (i.e., not including the accuracy of the training population on itself).*

The type ‘B’ correlations (Yamada, 1962), which measure the phenotypic expression of genetically similar individuals across environments (in this case, plots within the same site across different number of years) among the three populations is given in **Table 3**. All correlations of traits between populations were positive, with those between Las Tecas and the other two populations higher than those between Ganaderia and Malvinas (mean *r*^2^ values of 0.83 and 0.88, respectively, versus 0.79), though this relationship is not consistent across all phenotypes.

**Table 3:** Type ‘B’ correlation among three populations of cacao for six phenotypes.

Trait	Training population	Test population	
		Malvinas	Las Tecas
**Vegetative broom**	Ganaderia	0.909	0.999
	Malvinas	–	0.924
**Chirimoya pods**	Ganaderia	0.810	0.578
	Malvinas		0.907
**Cushion broom**	Ganaderia	0.996	0.947
	Malvinas	–	0.883
**Monilia pods**	Ganaderia	0.430	0.700
	Malvinas	–	0.804
**Healthy pods**	Ganaderia	0.863	0.942
	Malvinas	–	0.998
**Total fresh weight**	Ganaderia	0.722	0.880
	Malvinas	–	0.792

### Early WBD Phenotypic Selection

For one of three populations (Las Tecas) accessions were scored at the seedling stage on three dates to determine potential resistance to WBD before field trials. Of the total population of 569 test accessions (minus those removed from the analysis due to incomplete genotype data), 305 were scored as ‘Resistant’ (showing no sign of WB infection), 105 as ‘Partially Resistant’ (showing symptoms of WB on the first date but not subsequent dates) and 159 as ‘Susceptible’ (showing symptoms on all three dates which developed into brooms) but were nonetheless retained because the broom to stem ratio was smaller than 0.6 (see Materials and Methods). The mean actual values of the accessions in each seedling resistance category for the three WB diseases (vegetative broom, chirimoya pod, and cushion broom) as scored at maturity under field conditions are given in **Table 4**. Although the arithmetic means of the putatively resistant populations were lower than the susceptible in the case of vegetative brooms, they were actually higher in the case of chirimoya pods and cushion brooms. Nevertheless, the standard deviation of means was high in all cases, and the differences between the populations can be considered negligible.

**Table 4:** Mean values of three witches’ broom disease phenotypes observed at maturity grouped by their WB seedling phenotype score in a single population of cacao (Las Tecas).

Symptom Score^∗^	*N*	Vegetative broom	Chirimoya pod	Cushion broom
Resistant	305	0.79 ± 0.288	0.13 ± 0.114	0.24 ± 0.164
Partially RResistant	105	0.86 ± 0.256	0.14 ± 0.165	0.20 ± 0.202
Susceptible	59	0.78 ± 0.259	0.10 ± 0.146	0.24 ± 0.177

*^∗^ Out of three observation dates, accessions showing symptoms in all dates were marked ‘Susceptible,’ those in -2 dates were marked ‘Partially Resistant,’ and those showing no symptoms were marked as ‘Resistant.’*

### Selection Simulation

**FIGURE 6:**
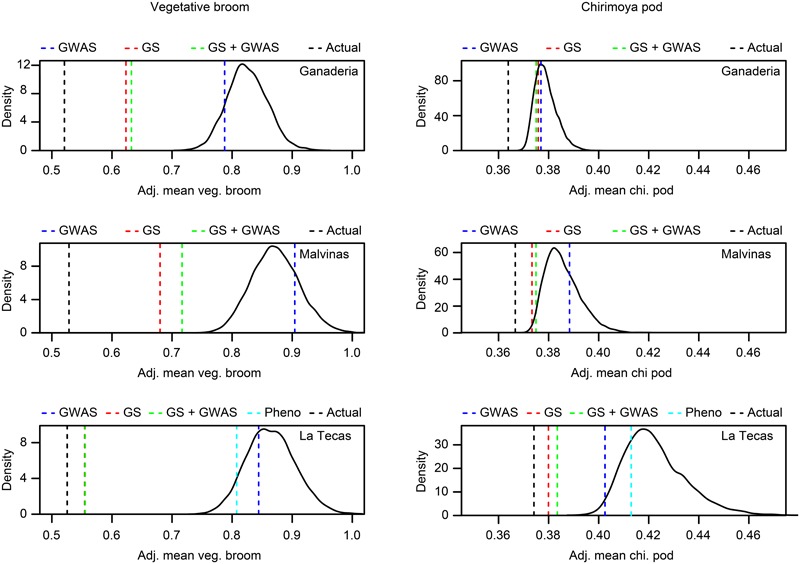
Simulated selection screen of two traits (vegetative broom and chirimoya pod) in three populations of cacao using three genetic prediction methods and one phenotypic method, compared against a random sampling of the populations. The predicted top ∼10% (40 individuals) performers for each phenotype from each population (‘Ganaderia,’ ‘Malvinas,’ ‘Las Tecas’) were selected using predictions from the training population (‘Las Tecas’), using three different methods (‘GWAS’ = ranking by sum of desirable GWAS-derived markers, ‘GS’ = ranking by genomic selection model GEBV, ‘GS + GWAS’ = ranking by genomic selection model GEBV with GWAS markers as fixed effects, ‘Pheno’ = phenotypic selection of seedlings for disease susceptibility (in Las Tecas only). Curve indicates the distribution of means from a 10,000-fold sampling of 40 random accessions from the training population. Lines indicate the position of the mean of the set selected by each method, including the actual top 10% selected by observed phenotypes. Sets outside of the random distribution are significantly different than the population mean at *P* < 0.0001.

**FIGURE 7:**
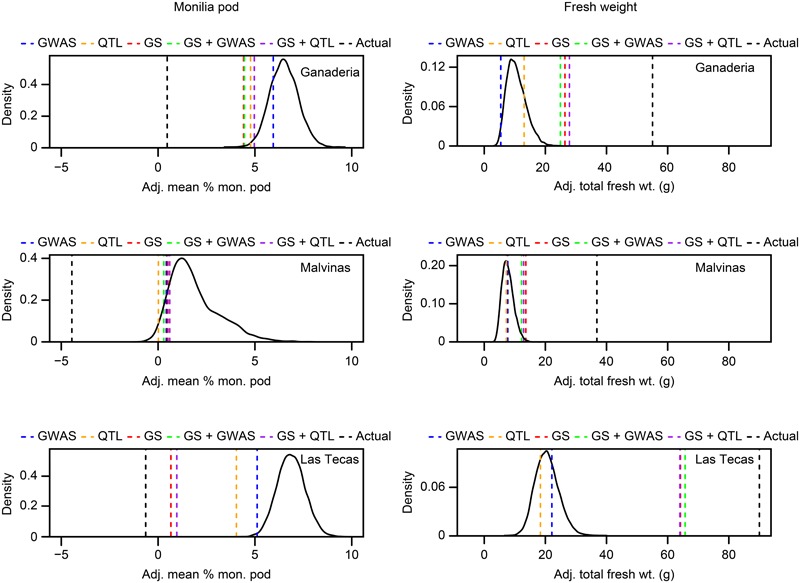
Simulated selection screen of two traits (monilia Pod, total fresh weight) in three populations of cacao using five genetic prediction methods, compared against a random sampling of the populations. The predicted top ∼10% (40 individuals) for each phenotype from each population (‘Ganaderia,’ ‘Malvinas,’ ‘Las Tecas’) were selected using predictions from the training population (‘Las Tecas’), using three different methods (‘GWAS’ = ranking by sum of desirable GWAS-derived markers, ‘QTL’ = ranking by sum of desirable biparental population QTL markers, ‘GS’ = ranking by genomic selection model GEBV, ‘GS + GWAS’ = ranking by genomic selection model GEBV with GWAS markers as fixed effects, ‘GS + QTL’ = ranking by genomic selection model GEBV with QTL markers as fixed effects). Curve indicates the distribution of means from a 10,000-fold sampling of 40 random accessions from the training population. Lines indicate the position of the mean of the set selected by each method, including the actual top 10% selected by observed phenotypes. Sets outside of the random distribution are significantly different than the population mean at *P* < 0.0001.

To simulate a screening of germplasm via genotyping, we used the data from the GWAS and GS of Las Tecas, which was not only the largest population of the three but also the one with the most years of phenotypic data available, to predict the top 40 individuals (∼10% selection intensity) performers in the Ganaderia and Malvinas populations. The top 40 individuals, as predicted by GWAS markers, QTL markers discovered in a related biparental population (Livingstone et al., 2017), GS and additional GS models that incorporated the GWAS/QTL markers as fixed effects, were compared to the phenotypic distribution of the entire population, as well as the actual top 10% of performers, for the traits vegetative broom, chirimoya pod and fresh weight. In the case of ‘ties,’ resulting in more than 40 individuals sharing the same top score, 40 were selected at random. The results (**Figures 6, 7**) show that GS was the most accurate selection method and suggests that the addition of markers as fixed effects had a negligible impact on prediction accuracy. Focusing solely on the Las Tecas population, early phenotypic selection gave a slight advantage in selecting for vegetative broom, but none for chirimoya pods. In addition, because so many accessions were scored as ‘Resistant’ in phenotypic scoring (305, 51% of the population) without any means for further discrimination, the level of resistance within the population would still depend largely on random chance.

## Discussion

### Population Structure

Although the three populations used in this study were not genetically dissimilar (**Figure 3**), some key differences existed between them. This was most apparent when observing the breakdown of LD (**Figure 4**), which remained much higher in one population than the other two. Part of this finding may be explained by ancestry: both Ganaderia and Las Tecas are dominated by ‘Wild’ and ‘Nacional’ types, respectively, while Malvinas is composed mostly of crosses among “known accessions.” Malvinas is more diverse in terms of ancestry distribution (**Figure 2**, **Table 1**, and **Supplementary Table S1**), but it is derived from long-cultivated varieties which likely have a higher degree of LD than their wilder counterparts, as has been described in cacao previously (Stack et al., 2015).

### GWAS Markers

Selection using small sets of markers associated with desired phenotypes is a more traditional approach to MAB, and is a viable option for many crops (Bouchez et al., 2002; Zhou et al., 2003; Fan et al., 2006; Kuchel et al., 2007). The use of GWAS has allowed molecular biologists to look beyond bi-parental crosses and closely interrelated populations to find robust markers across diverse individuals in numerous crops (Cockram et al., 2010; Kump et al., 2011; Migicovsky et al., 2016; Berdugo-Cely et al., 2017). In total, we found 18 SNPs significantly associated with disease phenotypes, and an additional 10 SNPs associated with productivity (fresh weight). Many of these markers occurred in areas identified in previous studies, as described below. A large number of disease markers occurred on chromosome 9 (**Figure 5** and **Supplementary Table S1**), known to be a ‘hot spot’ for WBD resistance, as well as for FPRD and BPR (*Phytophthora*; Brown et al., 2005; Lanaud et al., 2009; Fister et al., 2016; Royaert et al., 2016). It has been suggested that the source of this resistance may be related to the function of a Uveal Autoantigen with Coiled-coil domains and Ankyrin repeats (UACA) gene, which triggers cell apoptosis when DNA damage is detected (Royaert et al., 2016). Given that this molecular-level response would be effective against a wide range of fungal pathogens, it is perhaps not surprising that hits for both vegetative broom and monilia pod infection occur there. Another notable set of markers occur on a region of approximately 550 kbp in chromosome 1, where significant markers for chirimoya pod, cushion broom and monilia pod were found in the Malvinas population. No QTLs for traits specific to that region have been identified previously, though this has been identified as a region associated with resistance to *Phytophthora* diseases (Lanaud et al., 2009). The fact that neither of the other two populations had significant hits in this area suggests that their effectiveness outside of closely related germplasm may be limited. Finally, a region on the anterior of chromosome 10 was also somewhat enriched in markers, with hits for fresh weight and monilia pods (traits that show at least some correlation, see **Figure 1**) across all populations. Putative pathogen defense-related genes have been identified in the area (Lanaud et al., 2004; Brown et al., 2007), though they have not been widely reported.

Although many good candidate loci may have been identified by GWAS, it is important to note that few are shared across populations. This finding could be explained by several factors. First, although the three populations overall were not that different in their genetic structure, they were enriched differently in terms of either ‘Wild,’ ‘Known Accessions’ or ‘Nacional’ type crosses. The ‘Wild’ parents were observed to show tolerance against WBD (after a 2-year evaluation process in the germplasm collections) unlike the Nacional-type parents. This observation may suggest that resistance genes may have been distributed differentially among the three populations, hence the low repeatability of markers significantly associated with resistance. Even in the case that similar resistance alleles were present in the different populations, if they were inherited from different parents it is possible that the marker-allele association was not conserved, leading to population-specific markers (Biscarini et al., 2010). Furthermore, disease resistance is a complex and evolving trait and is more likely to be polygenic when considered over multiple years and environments (Lindhout, 2002). This is particularly true in cases such as our study, where multiple sources of diverse germplasm, each carrying its own (polygenic) resistance mechanisms, are introgressed. While efforts have been made to modify GWAS to be better able to handle polygenic traits (Segura et al., 2012), its main strength is identifying single markers with large effects, making its ability to robustly predict traits such as disease resistance limited.

### Genomic Selection

Unlike GWAS, GS is designed to be able to consider multiple markers when predicting phenotypes from genotypes. In general, our models had good predictive ability for some more heritable traits (i.e., vegetative brooms, total fresh weight). Although the Las Tecas population had a greater number of accessions and arguably higher quality phenotype data (based on 5 years instead of 3), models using it as a training population were not much higher in accuracy than those using the two smaller populations. However, it could predict phenotypes of the two smaller populations better than they could themselves. As the accuracy of GS models can depend heavily on the size of the training population (Zhong et al., 2009), this is not surprising.

### Early Phenotypic Screening of WBD Incidence

Early phenotypic selection of accessions is a common practice in cacao breeding that has been used effectively in the past (Surujdeo-Maharaj et al., 2003; Thévenin et al., 2010). In our study, the procedure did an adequate job of selecting accessions that were less likely to be susceptible to vegetative broom formation, though not other forms of WBD (chirimoya pods), but it was still much less effective than genomic selection (**Figure 6**). Much like selection by GWAS markers, this method could identify individuals who were extremely susceptible very easily, but it was unable to distinguish between plants that had moderate or high resistance. In our study, nearly half of the accessions tested had a nearly identical ranking (i.e., showing no signs of WBD at the seedling stage), making selections from this set only slightly better than random.

### Selection Simulation

Although both GWAS and GS offer different approaches to MAB, the ultimate test of these methods lies in their ability to be applied in an actual breeding situation. We decided to simulate an early-stage germplasm population screening wherein 10% of the accessions would be selected from a population based on their predicted performance from genotypic information. As a training population, we selected Las Tecas as it offered the largest number of accessions and the best-quality phenotype data. We then selected three traits that represented slightly different scenarios: vegetative broom, which had high GS prediction ability (0.477) and markers only moderately associated with the trait (i.e., with a GWAS *M*_eff_ – adjusted *P*-value at < 0.1 level, rather than below the typical 0.05 threshold) in the training population, chirimoya pods, which had three strongly associated single markers (GWAS *M*_eff_ – adjusted *P*-value < 0.05) but poor GS predictability (0.176), and total fresh weight, which had moderate values for both GS (0.391) and a single, strongly associated marker. Of the three models used to rank accessions (GWAS marker score, GS-predicted phenotype, GWAS marker Fixed Effect GS-predicted phenotype), none matched the ‘true’ value (i.e., the actual top ranked 40 individuals in Ganaderia and Malvinas for each trait), but it did reveal several important issues.

First, the selection by GWAS markers alone did not significantly improve selection for any trait in either population over what could be considered random chance selection. This is not altogether surprising, given that our prior GWAS analysis showed us that Las Tecas had no significant markers in common with the other two populations for those traits. However, even though there were more markers in common, it is still unlikely that GWAS would have improved the selection significantly, because at least two of the disease markers, the minor allele was associated with susceptibility rather than resistance. These types of markers would therefore be useful in identifying individuals with the poorest predicted performance, but in severe selection sweeps such as ours, would not contribute much to predicting individuals with above-average phenotypes.

Genomic selection, on the other hand, could select top performers much better, selecting a significantly better subset in vegetative broom in both populations, and a mean fresh weight in Ganaderia. The addition of GWAS or QTL markers as fixed effects provided little improvement to predictive ability and in some cases reduced it. Again, this finding is perhaps not entirely surprising, given our prior knowledge that the GWAS makers were unlikely to be applicable to the population, a caveat to this method (Bian and Holland, 2017). On the other hand, in a real selection sweep, it would not be unrealistic to assume that markers having significant associations in a genetically similar population would confer some level of phenotypic improvement.

Disease resistance in crops is often thought of as a qualitative trait, with genotypes falling into categories of ‘Resistant’ or ‘Susceptible’ due to a small number of genetic loci. For this reason, breeders often approach the selection of disease-resistant germplasm as being well-suited to marker-assisted selection (MAS) while leaving traits thought to be more quantitative in nature (e.g., yield) to complex whole-genome techniques such as GS. We have demonstrated that for plant diseases with no single, large-effect QTLs, GS may be a more effective selection method to screen for disease resistance. This efficacy was also recently demonstrated by our team on a smaller population in Central America (Navarro et al., 2017).

It should be noted that although GS was the most efficient technique at selecting resistant germplasm, it does have limitations, including a higher cost of genotyping than single-marker testing and the need for phenotypic data from a training population. In this way, GS can be thought of as a tool most useful in a mature breeding program for which ample data have already been generated. Single marker selection and early phenotypic evaluation, on the other hand, are most useful at the early stages of germplasm development, where the elimination of very susceptible types can be eliminated from the pool before resources are spent on field-testing them. Ultimately, the right tool for the right job will lead to the best results when combatting *Moniliophthora* spp. diseases in cacao.

## Conclusion

Resistance to *Moniliophthora* diseases in cacao is an important trait that may be improved via MAB. In a study of three related populations of cacao, several markers were identified for disease resistance and productivity via GWAS, but these were not consistent across populations, perhaps due to their distinctive germplasm structure. Genomic selection was used to predict phenotypes using each site as a training population for the remaining two; prediction accuracies varied between training populations and traits. Finally, a simulation of a screening selection was made wherein the top 10% of individuals in two populations were made with the GWAS marker data and GS using the largest population as a training population. The predictive accuracy was much higher when using GS than single-marker selection or early phenotypic selection, which demonstrates its effectiveness as a technique for selecting superior disease-resistant germplasm in tropical perennials.

## Author Contributions

MM, AN, GM, CS, SG, GD, ZM, and JM performed the genotyping, population structure, phenotypic selection, and genetic mapping analyses. GP, WS, DS, IS, GM, OT, and JM selected the clones for trials and coordinated phenotypic data recording and curation. FA, SM, and JM conceived of and conducted the experiments. MM, SM, and JM wrote the manuscript.

## Conflict of Interest Statement

The authors declare that the research was conducted in the absence of any commercial or financial relationships that could be construed as a potential conflict of interest.

## Supplementary Material

TABLE S1 Dialell crosses per genetic types: **(a)** Wild Types, **(b)** Known Clones, and **(c)** Nacional. Number refers to the total crosses performed per combination.

TABLE S2 Adjusted means of seven phenotypic traits (count of vegetative brooms, count of cherimoya pods, count of cushion brooms, count of total pods, percent of healthy pods, total pod fresh weight) by accessions of three populations of cacao.

TABLE S3 Genomic position, *P*-value and variance explained (*r*^2^) of SNPs significantly associated with five phenotypes among three populations of cacao.
