# Supplementary material for: Prediction of Cacao (Theobroma cacao) Resistance to Moniliophthora spp. Diseases via Genome-Wide Association Analysis and Genomic Selection
Source: Front Plant Sci. 2018 Mar 20;9:343. doi: 10.3389/fpls.2018.00343 (PMC5890178; doi:10.3389/fpls.2018.00343)
Supplement: TABLE S1 — Dialell crosses per genetic types: (a) Wild Types, (b) Known Clones, and (c) Nacional. Number refers to the total crosses performed per combination. [file Table_1.PDF]

Table S1: Dialell Crosses per genetic types: a) Wild Types, b) Known Clones and C) Nacional. Number refer to the total crosses performed per combination

a) **Wild Types**

	Paternal														
**Maternal**	**AMAZ-11**	**AMAZ-14**	**CUR-3**	**EBC-148**	**IMC 57**	**LCT-368**	**LCT-37**	**LCT-46**	**PA 107**	**TAP-10**	**TAP-12**	**TAP-3**	**TAP-6**	**TIP-1**	**UNAP-2**
**AMAZ-11**			1	2		2				1	1	1	1	1	1
**AMAZ-14**			1	2		2			1		1			1	1
**CCN-51**	1	1	1	2	1	2	1	1	1	1	1	1	1	1	1
**CUR-3**				2		3								1	1
**EBC-148**						1									
**LCT-37**	1	1	1	1		1						1		1	1
**LCT-46**		1	1				1			1	1			1	1
**TAP-10**			1	2		1						1		1	1
**TAP-12**			1	1		1								1	1
**TAP-3**			1	2		2					1		1	1	1
**TAP-6**			1	2		2								1	1
**TIP-1**				1		2			1						
**UNAP-2**				2		2			1					1	

b) **Known Clones**

	Paternal																			
**Maternal**	**2057**	**2367**	**2416**	**A 2076**	**A 2126**	**A 2462**	**A 2634**	**A 2699**	**A 2748**	**A645**	**B60**	**CCAT-46-88**	**CCN-51**	**D147**	**EBC-148**	**EET-400**	**EET-416**	**EET-446**	**EET-450**	**EET-451**
**AMAZ-11**					1	1	1	1	1		1			1						
**AMAZ-14**				1					1	1	1			1						
**CCAT-46-88**													2							
**CCN-51**	1	1		1	1	1				2	2	1		2			1	1	2	1
**CUR-3**														1						
**EET-233**	1	1		1					1	2	1			1						
**EET-387**	1		1							1	3			1	1		1			
**EET-446**																1				
**EET-58**	1		1								1									
**SIL-1**	1		1		1						1			1						
**SNA-0707**										1										
**SNA-0708**				1						1	1									
**SNA 0405**													2							

**SNA 0512**													2							
**TAP-3**							1			1	1			1						
**TAP-6**								1		1	1			1				1		
**TIP-1**					1				1		1									
**UNAP-2**					1		1			1	1									

c) **‘Nacional Types**

	Paternal													
**Maternal**	**A 2076**	**A 2699**	**CCAT-18-58**	**CCAT-46-88**	**CCAT-49-98**	**CCAT 1119**	**CCN-51**	**D147**	**EB-10-13**	**EB-22-37**	**EET-544**	**EET-95**	**SNA-0707**	**SNA-0708**
**Brisas-13**			1						1	1			1	1
**Brisas-16**				1										
**Brisas-3**			1											
**Brisas-30**			1							1				
**CCAT-18-58**	1							1			1	1		
**CCAT-46-68**							1							
**CCAT-49-98**		1												
**CCN-51**					1	1								
**Gloria-1**			1	1					2	2			1	1
**Gloria-17**				1						1			1	1
**Gloria-3**			1	1					1	1			1	1
