# Supplementary material for: Prediction of Cacao (Theobroma cacao) Resistance to Moniliophthora spp. Diseases via Genome-Wide Association Analysis and Genomic Selection
Source: Front Plant Sci. 2018 Mar 20;9:343. doi: 10.3389/fpls.2018.00343 (PMC5890178; doi:10.3389/fpls.2018.00343)
Supplement: TABLE S3 — Genomic position, P-value and variance explained (r2) of SNPs significantly associated with five phenotypes among three populations of cacao. [file Table_3.pdf]

Population	Trait	Chr.	Position (BP)	*p*-value	Marker R^2
Malvinas	Chirimoya Pod	1	5425381	6.03×10^{-11}	0.131
Malvinas	Cushion Broom	1	5506394	5.87×10^{-6}	0.065
Malvinas	Monilia Pod	1	5711689	2.25×10^{-5}	0.058
Ganaderia	Fresh Weight	1	34352267	5.66×10^{-7}	0.075
Tecas	Chirimoya Pod	1	34741393	6.32×10^{-7}	0.050
Ganaderia	Fresh Weight	2	1567531	9.35×10^{-7}	0.073
Tecas	Cushion Br	2	41557641	1.09×10^{-5}	0.040
Ganaderia	Fresh Weight	3	19309282	1.03×10^{-5}	0.060
Malvinas	Monilia Pod	3	27634037	2.62×10^{-6}	0.069
Ganaderia	Fresh Weight	3	29600619	3.32×10^{-6}	0.066
Malvinas	Monilia Pod	5	36110563	4.71×10^{-7}	0.079
Ganaderia	Cushion Br.	6	23401812	9.03×10^{-8}	0.087
Ganaderia	Fresh Weight	7	6470480	3.28×10^{-6}	0.066
Malvinas	Chirimoya Pod	7	15116846	1.44×10^{-8}	0.099
Tecas	Cushion Br	7	17120099	1.19×10^{-7}	0.056
Ganaderia	Vegetative Broom	8	1534915	3.66×10^{-6}	0.065
Ganaderia	Fresh Weight	8	12621160	9.08×10^{-6}	0.060
Malvinas	Monilia Pod	9	2233636	5.39×10^{-12}	0.145
Ganaderia	Fresh Weight	9	6870452	7.83×10^{-8}	0.086
Tecas	Chirimoya Pod	9	7802949	3.03×10^{-7}	0.053
Tecas	Chirimoya Pod	9	8630113	4.22×10^{-6}	0.043
Ganaderia	Vegetative Broom	9	14800629	3.06×10^{-6}	0.066
Malvinas	Monilia Pod	9	16273895	1.43×10^{-10}	0.126
Tecas	Monilia Pod	9	17023030	8.32×10^{-6}	0.041
Ganaderia	Fresh Weight	9	19161589	1.39×10^{-5}	0.058
Ganaderia	Vegetative Broom	9	27786422	4.22×10^{-6}	0.065
Tecas	Fresh Weight	10	1207822	2.46×10^{-6}	0.045
Malvinas	Monilia Pod	10	3489346	5.36×10^{-8}	0.091
Ganaderia	Fresh Weight	10	3902074	4.97×10^{-6}	0.064
